# Rats with congenital hydronephrosis show increased susceptibility to renal ischemia‐reperfusion injury

**DOI:** 10.14814/phy2.14638

**Published:** 2020-11-18

**Authors:** Reinis Vilskersts, Karlis Vilks, Melita Videja, Helena Cirule, Olga Zharkova‐Malkova, Eduards Sevostjanovs, Maija Dambrova, Edgars Liepinsh

**Affiliations:** ^1^ Latvian Institute of Organic Synthesis Riga Latvia; ^2^ Rigas Stradins University Riga Latvia; ^3^ University of Latvia Riga Latvia

**Keywords:** hydronephrosis, pharmacokinetics, renal ischemia‐reperfusion, ultrasonography

## Abstract

Many drug candidates have shown significant renoprotective effects in preclinical models; however, there is no clinically used effective pharmacotherapy for acute kidney injury. The failure to translate from bench to bedside could be due to misleading results from experimental animals with undetected congenital kidney defects. This study was performed to assess the effects of congenital hydronephrosis on the functional capacity of tubular renal transporters as well as kidney sensitivity to ischemia‐reperfusion (I‐R)‐induced injury in male Wistar rats. Ultrasonography was used to distinguish healthy control rats from rats with hydronephrosis. L‐carnitine or furosemide was administered, and serial blood samples were collected and analyzed to assess the effects of hydronephrosis on the pharmacokinetic parameters. Renal injury was induced by clamping the renal pedicles of both kidneys for 30 min with subsequent 24 hr reperfusion. The prevalence of hydronephrosis reached ~30%. The plasma concentrations after administration of L‐carnitine or furosemide were similar in both groups. I‐R induced more pronounced renal injury in the hydronephrotic rats than the control rats, which was evident by a significantly higher kidney injury molecule‐1 concentration and lower creatinine concentration in the urine of the hydronephrotic rats than the control rats. After I‐R, the gene expression levels of renal injury markers were significantly higher in the hydronephrotic kidneys than in the kidneys of control group animals. In conclusion, our results demonstrate that hydronephrotic kidneys are more susceptible to I‐R‐induced damage than healthy kidneys. Unilateral hydronephrosis does not affect the pharmacokinetics of substances secreted or absorbed in the renal tubules.

## Graphical abstract

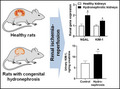

## 1 INTRODUCTION

Acute kidney injury (AKI) is defined as an abrupt loss in renal function and may be caused by various clinical conditions (Fortrie et al., 2019). The most common causes of AKI include decreased renal blood flow, ischemia, or sepsis (Farrar, 2018; Ronco et al., 2019). AKI occurs in approximately 10%–15% of patients admitted to the hospital, while its incidence has been reported to be more than 50% of patients in intensive care (Ronco et al., 2019). AKI is independently associated with a high risk of mortality and progressive deterioration of renal function. Furthermore, recent studies have suggested that AKI is also a risk factor for the development of other diseases (Fortrie et al., 2019; Zuk & Bonventre, 2016).

Despite continuing advances in the field of renal medicine, to date, there is no clinically used effective pharmacotherapy to decrease ischemia‐reperfusion (I‐R)‐induced kidney damage (O'Kane et al., 2019; Zuk & Bonventre, 2016). Thus, the search for new renoprotective substances is still important. For decades, various preclinical in vivo models with different modifications have been used to study the pathogenic mechanisms of ischemic AKI and to search for novel renoprotective substances (Wei & Dong, 2012). Nevertheless, the translation of new therapies to patients is very poor, and despite significant renoprotective properties in rodents, many drug candidates have failed to show significant protective effects in clinical trials (O'Kane et al., 2019). The failure to translate from the bench to the bedside has been attributed, in part, to the more complicated architecture of human kidneys than animal kidneys, to differences in the primary endpoints between clinical and animal studies and poor‐quality and irreproducible preclinical results (O'Kane et al., 2019). Indeed, some recent studies have claimed that a major portion of published results are not reproducible (Baker & Penny, 2016; Begley & Ioannidis, 2015). There are several possible explanations for the irreproducible results, such as inadequate experimental design, faulty randomization, statistical and reporting issues, and the lack of standardization of animal experimentation (Alstrup & Sonne, 2019). Thus, undetected congenital kidney defects, in part, could also be the reason for the irreproducible results and poor translatability from the bench to the bedside.

It has been claimed that developmental defects of the kidney are uncommon in rats. Cortical, medullary, and/or papillary cysts and hydronephrosis are the two most frequently observed developmental defects in rats (Seely et al., 2018). Generally, hydronephrosis occurs at a low incidence (<2%–6%) in most rat strains, and an increased incidence can be obtained by selective breeding of hydronephrotic animals (Van Winkle et al., 1988). Hydronephrosis is characterized by the dilatation of the renal pelvis, loss of renal medullary tissue, and microscopically by the atrophy of the renal tubules, glomeruli, and pelvic urothelium (Zhou et al., 2003), although some studies have demonstrated normal morphology in hydronephrotic kidneys (Friedman et al., 1979). More often, hydronephrosis is unilateral and affects the right side and occurs more frequently in male rats than female rats (Seely et al., 2018). Experimental hydronephrosis in rats can be induced by the ligation of the ureter (Gobe & Axelsen, 1987). Previous studies have shown that hydronephrosis, congenital, or surgically induced, can promote the development of other diseases (Carlström et al., 2006; Seely et al., 2018), can alter the functioning of other organs (Arnold et al., 2011; Marsh et al., 2007) and may be responsible for different responses to a given treatment (Steinhausen et al., 1987).

This study was conducted to assess the effects of hydronephrosis on renal vulnerability to I‐R‐induced injury as well as the pharmacokinetic parameters of substances that are transported via the tubular membrane of the nephron.

## 2 ANIMALS AND METHODS

Eighty‐six 8‐ to 9‐week‐old male Wistar rats (strain: RccHan:WIST) were obtained from Envigo (The Netherlands) in two separate shippings (the first group, *n* = 46 and the second group, *n* = 40). Animals from the first group were born approximately in November and animals from the second group were born approximately in March‐April. After the arrival, the experimental animals were housed under standard conditions (21‐23°C, 12‐hr light/dark cycle, relative humidity 45%–65%) with unlimited access to food (R3 diet; Lactamin AB, Kimstad, Sweden) and water. The experimental procedures were performed in accordance with the guidelines of the European Community as well as local laws and policies, and the procedures were approved by the Latvian Animal Protection Ethical Committee of the Food and Veterinary Service, Riga, Latvia. All data involving animals were reported in accordance with the ARRIVE guidelines (Kilkenny et al., 2010).

**Figure 1:**
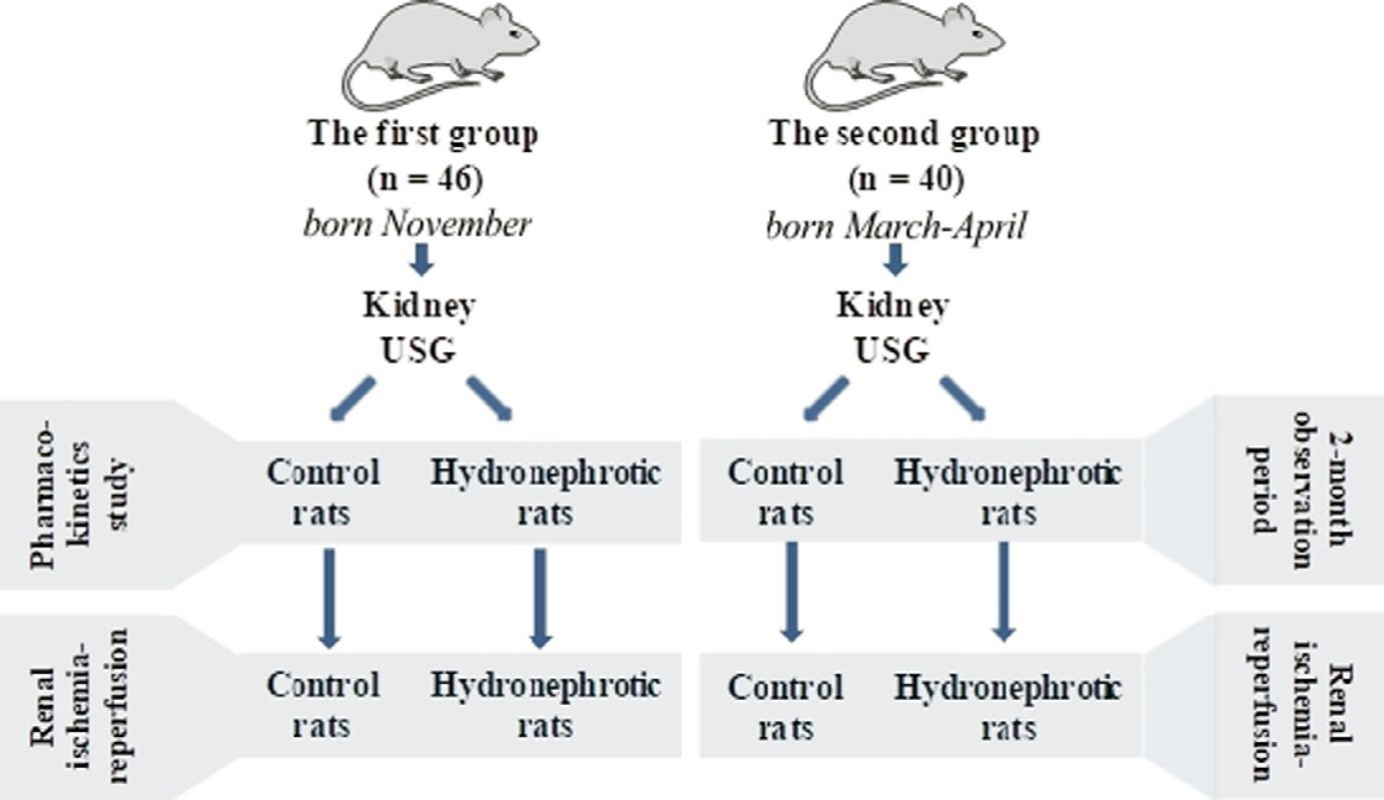
Schematic representation of study design

Schematic design of the experiments is displayed in Figure 1. Rats were adapted to the new conditions for 2 weeks before the beginning of any experimental procedures. After the adaptation period, the kidneys of the animals were examined using ultrasonography as described further. Animals with hydronephrosis (uni‐ and bilateral) were included in the hydronephrosis group, and a similar number of animals with normal kidneys were used as the control group. Only animals with unilateral hydronephrosis from the first group were subjected to a pharmacokinetics study (*n* = 5 per group). Animals with bilateral hydronephrosis were not included in the pharmacokinetics study as we had only four rats with bilateral hydronephrosis and it was not sufficient to comprise a group. Two 2 weeks later animals with uni‐ and bilateral hydronephrosis were subjected to renal I‐R (*n* = 12 per group). In addition, renal ultrasonograpy and 24‐hr urine samples with a 2‐month interval were collected from the second group of animals with unilateral hydronephrosis to estimate creatinine clearance (*n* = 6 per group), and then, the animals were subjected to renal I‐R (*n* = 6 per group) as described in detail further.

Determination of L‐carnitine, furosemide, blood urea nitrogen (BUN), and creatinine in the rat plasma; measurement of kidney injury markers and creatinine in urine; induction of renal I‐R injury and gene expression analysis were performed by scientific staff blinded to the experimental groups.

### 2.1 Ultrasonography

Experimental animals at the age of 10–11 weeks were anaesthetized with 2%–3% isoflurane dissolved in oxygen. The flow rate of the mixture of isoflurane and oxygen during ultrasonography was 2 L/min. After the onset of anesthesia, the region above the kidneys on the back and flanks was shaved, and the remaining fur was removed with commercially available depilation cream (Veet™). Optixcare eye lube was applied to the eyes to prevent corneal dryout and damage during the procedure. Kidney structures were analyzed in the transverse scan plane using a Philips iE33 ultrasonograph (Philips Healthcare, Andover, USA) equipped with a linear L15‐7io transducer.

### 2.2 Pharmacokinetics study

For analysis of the effects of unilateral hydronephrosis on the functional capacity of renal transporters in nephron tubules, L‐carnitine, or furosemide was administered subcutaneously at doses of 200 or 50 mg/kg, respectively. L‐Carnitine or furosemide were administered to rats when they were 11–12 or 13–14 weeks old, respectively. Both substances were used as they both are transported via transport proteins in renal tubule (Hasegawa et al., 2007; Stieger et al., 1995; Tamai et al., 1998), which may be affected by hydronephrosis (Zhou et al., 2003). Plasma concentrations of the compounds were assessed before treatment (only for L‐carnitine) and 15, 30, 60, 120, and 240 min after the administration of L‐carnitine or furosemide. Experiments were carried out after a 2‐week washout period to ensure that the previously administered substance was fully eliminated from the body.

### 2.3 Determination of L‐carnitine and furosemide in rat plasma by UPLC‐MS‐MS

Determination of L‐carnitine in plasma samples was performed by ultra‐performance liquid chromatography‐tandem mass spectrometry (UPLC‐MS‐MS) using the positive ion electrospray mode as described previously (Dambrova et al., 2008) with slight modifications.

Quantitative determination of furosemide in the rat plasma was performed by the UPLC‐MS‐MS method. Waters Acquity UPLC chromatograph was coupled to a Micromass Quattro Micro tandem mass spectrometer. Chromatographic separation was achieved on a Waters Acquity UPLC BEH C18 column (2.1 × 50 mm, 1.7 µm). The mobile phase consisted of a water and acetonitrile gradient. The acetonitrile composition was increased from 15% to 98% in 2.5 min, the total run time was 6 min, the flow rate was 0.25 ml/min, the column temperature was 30°C and the injection volume was 5 µl.

The mass spectrometer was operated in the negative ionization electrospray mode with capillary voltage −3.0 kV, source temperature 120°C, desolvation temperature 400°C, desolvation gas flow 800 L/h and cone gas flow 50 L/h. The sum of two MRM transitions was used for quantification: 329.1 → 205.1 (cone 20 V, collision 20 eV) and 329.1 → 285.0 (cone 20 V, collision 12 eV).

The concentrations of furosemide were measured against a six‐point calibration curve. Calibration standards were prepared by spiking the rat plasma in a concentration range of 1.56–200 µg/ml. Extraction of furosemide from the rat plasma was performed by deproteinizing 20 µl of sample with 300 µl of acetonitrile/methanol (3:1, v/v). The samples were vortexed and centrifuged at 10,850 *g* for 10 min. Two hundred microliter of supernatant was diluted with 600 µl of water and used for LC‐MS‐MS analysis.

### 2.4 Creatinine clearance

For determination of the effects of hydronephrosis on the glomerular filtration rate, the experimental animals from the second group at the ages of 10–11 and 18–19 weeks were housed in metabolic chambers to collect 24‐hr urine. Plasma samples were prepared from blood collected from the tail vein shortly before transferring the rats to metabolic cages. Plasma and urine creatinine concentrations and BUN were measured using commercially available kits (Instrumentation Laboratory). Creatinine clearance was calculated using the following formula:

$$\text{Creatinine clearance}=\frac{\text{Urinary creatinine}\left(\frac{\text{mg}}{\text{dl}}\right)\times \text{Urine volume}\left(\frac{\text{ml}}{h}\right)}{\text{Plasma creatinine}\left(\frac{\text{mg}}{\text{dl}}\right)}$$

### 2.5 Renal I‐R model

Rats from the first group were subjected to the renal ischemia‐reperfusion at the age of 15–16 weeks and rats from the second group at the age of 19–20 weeks. The experimental animals were anaesthetized using 2%–3% isoflurane dissolved in oxygen. The flow rate of the mixture of isoflurane and oxygen during the renal ischemia‐reperfusion was 2 L/min. After the onset of anesthesia, the animals received subcutaneous buprenorphine and benzylpenicillin at doses of 50 µg/kg and 150 mg/kg, respectively. The region above the kidneys on the back was shaved and disinfected with 5% iodine solution in ethanol. Later, iodine was cleaned from the skin using 70% ethanol. The animal was placed on a far infrared warming pad, and a rectal thermometer was inserted into the rectum. If needed, the animal was heated using an infrared lamp. The body temperature was maintained in the range of 36.6–37.2°C during the whole procedure. Surgery was not started until the body temperature was stabilized at the set‐point.

The rat was turned on the right side, and the skin and muscle layer on the left flank side was cut open along the back to expose the left kidney. The kidney was pushed out from the abdominal cavity with sterile cotton swabs to expose the renal pedicle, which was cleaned from surrounding fatty tissues. After cleaning the renal pedicle, the left kidney was returned to the abdominal cavity. The right renal pedicle was prepared by a similar surgical procedure. After cleaning the pedicle, the right kidney was returned back to its original position in the abdominal cavity. The rat was covered and allowed to adapt for 10 min. After the adaptation period, the right kidney was pushed out using sterile cotton swabs, and the renal pedicle was occluded using Schwartz Micro Serrefine. The pedicle of the left kidney was occluded in a similar manner. After vessel occlusion, both kidneys were returned to the abdominal cavity. The occlusion time (30 min) for each kidney was recorded separately. Reperfusion was induced by removing the clamp from the renal pedicle. Successful occlusion was visually confirmed by renal cyanosis and the dark blue color of the kidneys, and reperfusion was visually confirmed by a return to the normal color after removal of the clamp. A 4‐0 reabsorbable suture was used to close the muscle layer, followed by closure of the skin wound with rat wound clips. Immediately after wound closure, 1 ml of warm (37°C) sterile saline was given subcutaneously to each rat. The animal was then kept in a cage on a heating pad until it regained full consciousness. Four hours after the first injection of buprenorphine, a second dose (50 µg/kg) was administered. The body core temperature was measured just before decapitation to exclude animals with infection. Twenty‐four hours after the initiation of reperfusion, blood and urine samples were prepared from the first group of the animals to quantify creatinine and BUN in the plasma and creatinine and kidney injury molecule‐1 (KIM‐1) and neutrophil gelatinase‐associated lipocalin (NGAL) in the urine using commercially available kits from R&D Systems and AbCam, respectively. The second group of the animals was sacrificed by decapitation 24 hr after the initiation of reperfusion, and kidney tissue samples were collected for qPCR analysis in liquid nitrogen and kept at −80°C until further analysis.

### 2.6 mRNA isolation and qPCR analysis

Total RNA from the kidney tissues was isolated using TRI reagent (Sigma‐Aldrich), and first‐strand cDNA synthesis was carried out using a High Capacity cDNA Reverse Transcription Kit (Applied Biosystems™, Foster City, CA) following the manufacturer's instructions. qPCR analysis of gene expression was performed by mixing SYBR® Green Master Mix (Applied Biosystems™) with synthesized cDNA and forward and reverse primers. Primers were designed using the Primer‐BLAST tool (Ye et al., 2012) and are listed in Table S1. Reactions were run on a Bio‐Molecular Systems MIC qPCR Cycler according to the manufacturer's protocol and using the following conditions: 95°C for 10 min, (95°C for 15 s, 60°C for 60 s) (60 cycles), and 95°C for 60 s, followed by melt curve analysis at 72–95°C, 0.3°C/s. The relative expression levels for each gene were calculated with the ΔΔCt method and normalized to the expression of valosin‐containing protein (VCP).

### 2.7 Histopathological kidney examination

A portion of the tissues from each kidney was removed and fixed in 10% neutral phosphate‐buffered formalin. The fixed tissues were soaked for 24 hr in 30% sucrose solution and then embedded in OCT freezing media and frozen in −20°C. Several 10‐μm thick tissue sections were prepared and stained with hematoxylin and eosin.

Occlusion (20–45 min) of renal blood vessels with subsequent 24 hr reperfusion does not alter morphology of nephron corpuscle (Dong et al., 2019). The proximal tubule is the most vulnerable part of nephron to injury, due to its high rates of oxygen consumption and relative paucity of endogenous antioxidant defenses (Chevalier, 2016). Therefore in the renal sections we evaluated only the severity of tubular injury as described previously with slight modifications (Takaori et al., 2016). Tubular injury was scored semiquantitatively by an employee blinded to the animal groups. The employee examined 10 cortical fields at ×200 magnification. Tubular injury was defined as tubular dilation, tubular atrophy, tubular cast formation, vacuolization, degeneration, and sloughing off of tubular epithelial cells or loss of the brush border and thickening of the tubular basement membrane. The tubules were evaluated according to the following scoring system: 0 = no tubular injury; 1 = 10% tubules injured; 2 = 11%–25% tubules injured; 3 = 26%–50% tubules injured; 4 = 51%–74% tubules injured; and 5 = 75% tubules injured.

### 2.8 Data analysis

The data are expressed as the mean ± *SEM*. Statistical calculations were performed using Prism 8.3.1 software (GraphPad, San Diego, CA, USA). A paired *t* test was performed to compare the mean values of the cross‐sectional area of the renal pelvis, BUN, plasma creatinine, urine creatinine, and creatinine clearance before and after the 8‐week observation period. For comparison of the gene expression levels in the kidneys, one‐way ANOVA followed by Tukey's multiple comparison test as a post hoc test was used. The statistical significance between all other parameters was calculated using unpaired Student's *t* test or Mann–Whitney test depending on the data distribution which was calculated using Kolmogorov–Smirnov test. A two‐sided *p* < .05 was considered significant.

## 3 RESULTS

**Figure 2:**
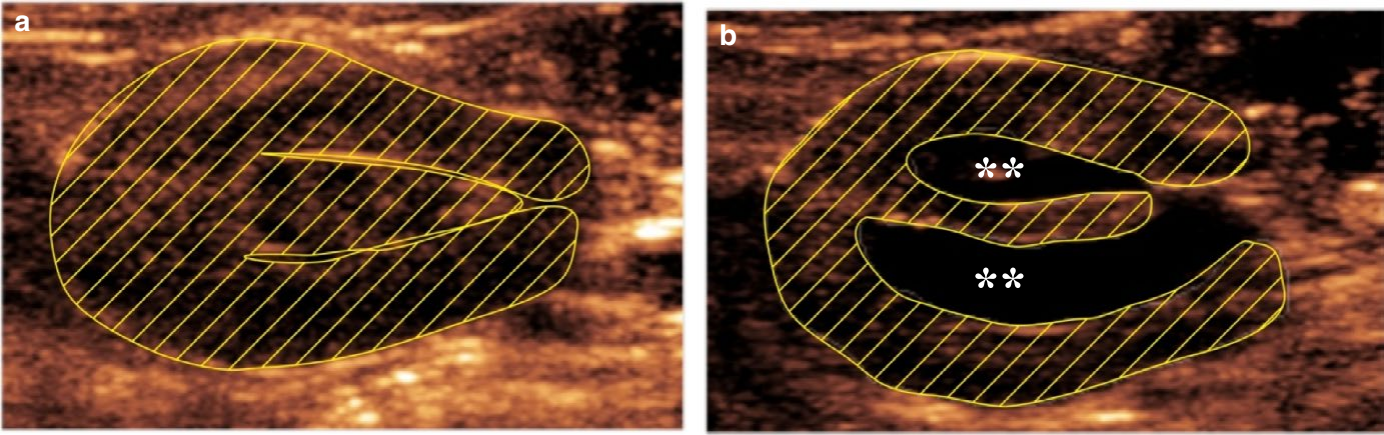
Illustrative ultrasonography images of a normal (a) and hydronephrotic (b) right kidney. Pictures were obtained from the transverse scan plane. Striped zones denote kidney parenchymal tissues, ** denotes substantially increased renal pelvis

The prevalence of hydronephrosis in Wistar rats was determined using ultrasonography. The analysis of the obtained data revealed that nearly one‐fourth to one‐third (26%–28%) of the animals in both groups had a dilated renal pelvis (Figure 2, Table 1). Most of the hydronephrotic animals (55%–67%) had an increased renal pelvis of the right kidney, and there were only two animals with left‐sided hydronephrosis. The remaining animals (~30%) had bilateral hydronephrosis. The area of renal pelvis was normalized to the area of the kidney as the size of the kidney may change over the time. The average area of the renal pelvis in the transverse sections of the right kidney in the hydronephrotic animals from both groups (*n* = 21) was 16 ± 1%. In the control group animals, the renal pelvis was too small for appropriate quantification and comprised <1% of area of the transverse section of right kidney (Figure 2). The degree of hydronephrosis varied between the animals, and in nearly half of the animals, the area of the renal pelvis comprised >10% of the area of the right kidney transverse plain. In the other animals, the dilatation of the right renal pelvis was not as pronounced and comprised less than 10% of the area of the right kidney transverse plain (Table 1).

**Table 1:** Prevalence and level of hydronephrosis in male Wistar rats

	Group I animals (*n* = 46)	Group II animals (*n* = 40)
Animals with hydronephrosis	12 (26%)	11 (28%)
Unilateral right sided	8 (67%)	6 (55%)
Unilateral left sided	0 (0%)	2 (18%)
Bilateral	4 (33%)	3 (27%)
Renal pelvis area >10% of the right kidney area	6 (50%)	4 (44%)
Renal pelvis area <10% of the right kidney area	6 (50%)	5 (56%)

**Figure 3:**
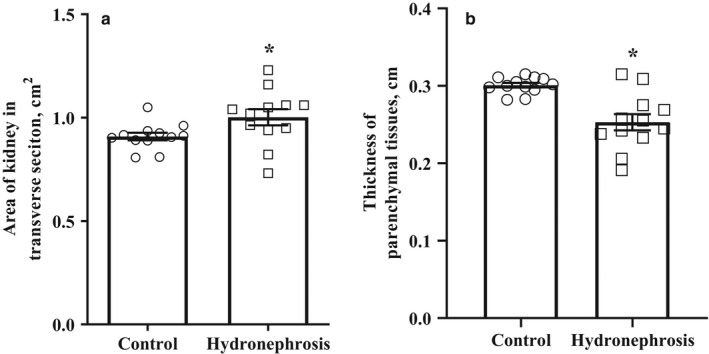
Anatomical dimensions of the right kidney of the first group of animals at the age of 10–11 weeks. Kidney area in the transverse scan plane (a) and the thickness of parenchymal tissues in the transverse scan plane (b). Hydronephrotic kidneys had larger transverse section area and thinner layer of parenchymal tissues than the control kidneys. The results are represented as the mean ± *SEM*of 12 animals in each group. *p < .05 Unpaired Student's*t*test versus normal right kidney from the control group animals

In addition to the enlarged renal pelvis, the animals with hydronephrosis had a larger renal area of the transverse section than the control animals (Figure 3a). The areas of the transverse kidney sections in the control and hydronephrotic animals were 0.9 ± 0.03 and 1.0 ± 0.04 cm^2^ (*p* < .05), respectively. Moreover the kidneys with hydronephrosis had thinner layer of parenchymal tissues than the control kidneys (Figure 3b). The parenchymal tissue thickness in the kidneys from the control and hydronephrotic animals was 0.3 ± 0.01 and 0.25 ± 0.03 cm, respectively.

**Figure 4:**
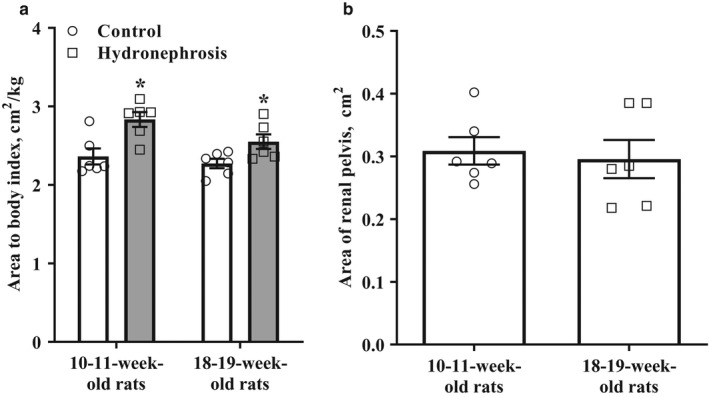
The dynamics of hydronephrosis development in the right kidney during the 2‐month observation period. During the 8‐week observation period, the area of kidney cross‐sectional area to body weight did not change, although the hydronephrotic kidneys were significantly larger than the healthy kidneys (a). The area of the renal pelvis of the hydronephrotic kidney did not increase during the 8‐week observation period (b). The results are shown as the mean ± *SEM*of 6 animals in each group. *p < .05 Unpaired Student's*t*test versus the normal right kidney from the control group animals

As shown in Figure 4a, the rats with hydronephrosis had a higher kidney cross‐section area to body weight index of the right kidney than the control rats. A significant difference in the kidney cross‐section area to body weight index was observed in 11‐ to 12‐week‐old rats and 19‐ to 20‐week‐old rats. The analysis of the cross‐sectional area of the renal pelvis of the hydronephrotic kidney (Figure 4b) revealed that hydronephrosis did not progress during the 2‐month observation period.

The analysis of plasma and urine samples revealed that the BUN, plasma, and urine concentrations of creatinine, 24‐hr urine output, and creatinine clearance were similar in the control and hydronephrotic animals at both studied time points (Table 2).

**Table 2:** Kidney functional parameters during the 8‐week observation period

	10‐ to 11‐week‐old rats		18‐ to 19‐week‐old rats	
	Control	Hydronephrosis	Control	Hydronephrosis
BUN, mM	6.2 ± 1.0	5.7 ± 1.6	6.8 ± 0.6	7.0 ± 0.5
Plasma creatinine, mg/L	55 ± 19	61 ± 18	21 ± 3	22 ± 4
Urine creatinine, mg/ml	0.7 ± 0.06	0.7 ± 0.09	0.9 ± 0.20	0.7 ± 0.10
24‐hr urine output, ml	15 ± 2	15 ± 3	19 ± 4	19 ± 5
Creatinine clearance, ml/h	12.5 ± 3.6	12.4 ± 6.4	29.1 ± 3.6	25.9 ± 5.9

Note

The results are shown as the mean ± *SEM* of six animals in the 10‐ to 11‐week‐old control and 18‐ to 19‐week‐old hydronephrotic animal groups and of five animals in the 10‐ to 11‐week‐old hydronephrotic and 18‐ to 19‐week‐old control group animals. One animal from each of the last two groups was excluded due to technical problems during plasma or urine analysis.

**Figure 5:**
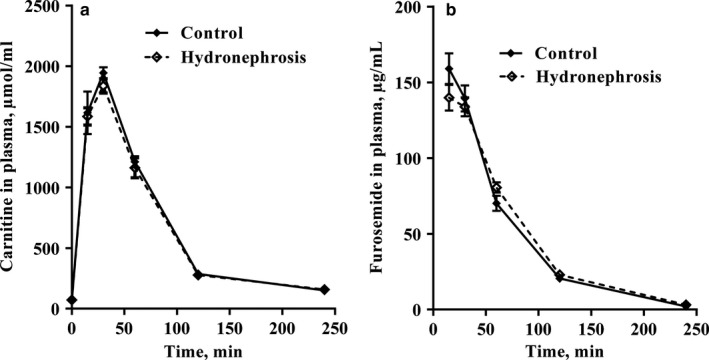
Effects of unilateral hydronephrosis on the L‐carnitine (a) and furosemide (b) concentrations in the plasma after a single subcutaneous administration. Unilateral hydronephrosis did not influence the pharmacokinetic parameters of L‐carnitine and furosemide. The results are shown as the mean ± *SEM*of 5 animals in each group

As shown in Figure 5, there were no differences in the plasma pharmacokinetic profiles of L‐carnitine (5a) or furosemide (5b) in the hydronephrotic and healthy rats. The maximal concentration of L‐carnitine in the plasma in both groups was observed approximately 30 min after administration, and L‐carnitine returned to its initial concentration after 4 hr. Similarly, there were no differences in the clearance of furosemide in both groups.

**Figure 6:**
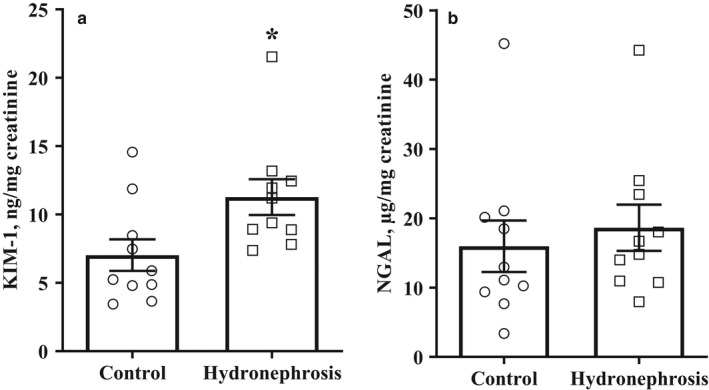
Effects of hydronephrosis on I‐R‐induced injury in the kidneys of 15–16 weeks old animals. Rats with hydronephrosis after renal I‐R had significantly higher concentrations of kidney injury marker‐1 (a) and slightly elevated neutrophil gelatinase‐associated lipocalin (b) concentrations in urine compared to healthy control group animals. The results are shown as the mean ± *SEM*of 10 animals in each group. *p < .05 Mann–Whitney test versus control group animals

Four animals, two from each group, were excluded from the analysis of results from the renal I‐R experiment due to a decreased body core temperature (one animal) or technical problems during surgery (three animals). After bilateral renal I‐R, the rats from the control and hydronephrosis groups had similar BUN levels in the plasma (Table 3). The animals in the hydronephrosis group had a 5 mg/L higher plasma creatinine concentration and a twofold significantly decreased creatinine concentration in urine compared to the animals in the control group. The analysis of kidney injury markers in the urine revealed that the animals with hydronephrosis had significantly higher KIM‐1 concentrations than the control animals (Figure 6a). The KIM‐1 concentrations in the urine of the control and hydronephrosis group animals were 7 ± 1 and 11 ± 1 ng/mg creatinine (*p* < .05), respectively. In addition, the urine concentration of NGAL was similar in both groups (Figure 6b). The NGAL concentrations in the control and hydronephrosis group animals were 16 ± 4 and 19 ± 3 ng/mg urine creatinine, respectively.

**Table 3:** BUN, plasma, and urine creatinine concentrations in 15‐ to 16‐week‐old rats 24h after the initiation of reperfusion

	BUN, mM	Plasma creatinine, mg/L	Urine creatinine, mg/L
Control	15 ± 2	14 ± 2	13 ± 3
Hydronephrosis	14 ± 2	19 ± 3	6 ± 1*

Note

The results are shown as the mean ± *SEM* of 10 animals in each group.

* *p* < .05 unpaired Student's *t* test versus the control group.

**Figure 7:**
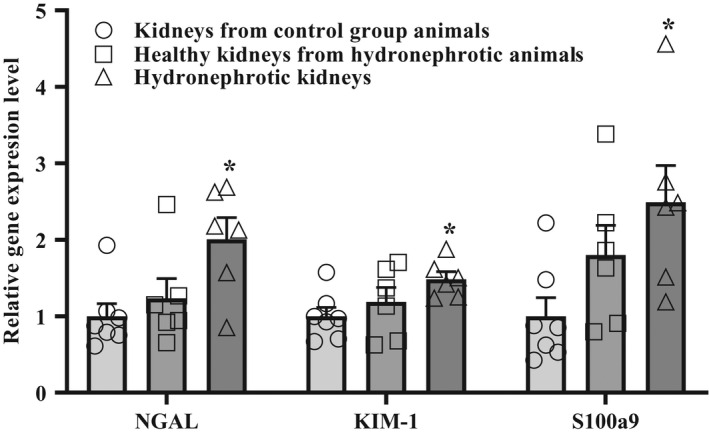
Effects of I‐R on NGAL, KIM‐1, S100a9 gene expression in the healthy and hydronephrotic kidneys. I‐R induced significant higher gene expression levels of NGAL, KIM‐1, and S100a9 in the hydronephrotic kidneys than in the kidneys of the control group animals at the age of 19–20 weeks. The results are shown as the mean ± *SEM*of 7 (4 right‐ and 3 left‐sided) kidneys from the control group animals and 6 right‐sided (hydronephrotic) and 6 left‐sided (healthy) kidneys from the animals with unilateral right‐sided hydronephrosis. *p < .05 one‐way analysis of variance followed by Tukey's multiple comparison test

As shown in Figure 7, renal I‐R induced significantly higher gene expression of NGAL, KIM‐1, and S100a9 in the hydronephrotic kidneys than the healthy kidneys from the control animals. In the rats with unilateral hydronephrosis, higher gene expression of NGAL, KIM‐1, and S100a9 was observed in the kidneys with hydronephrosis than in the healthy (left) kidneys; however, this difference was not significant.

**Figure 8:**
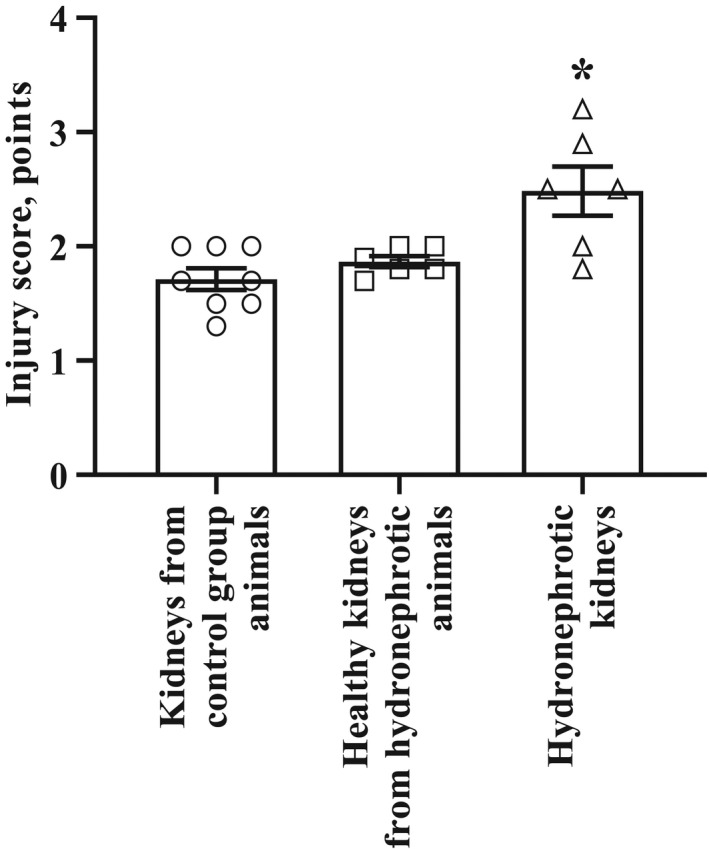
Morphological injury score after I‐R. Occlusion with subsequent reperfusion induced higher injury score in hydronephrotic kidneys than in kidneys of the control group animals. The results are shown as the mean ± *SEM*of 8 (4 right‐ and 4 left‐sided) kidneys from the control group animals and 6 right‐sided (hydronephrotic) and 6 left‐sided (healthy) kidneys from the animals with unilateral right‐sided hydronephrosis. *p < .05 one‐way analysis of variance followed by Tukey's multiple comparison test

The analysis of stained kidney sections revealed that the average injury score in control group was 1.7 ± 0.1 points. The kidney injury score after 30 min occlusion with subsequent 24 hr reperfusion was significantly higher in the hydronephrotic kidneys and reached 2.5 ± 0.2 points (Figure 8).

## 4 DISCUSSION

The results of this study demonstrate that hydronephrotic kidneys are more susceptible to renal I‐R‐induced injury than healthy kidneys; furthermore, the function of hydronephrotic kidneys is not altered or is fully compensated by the contralateral healthy kidney. In addition, our results demonstrate that the prevalence of hydronephrosis in Wistar rats can be much higher than claimed in the scientific literature or by animal breeders.

It is generally accepted that the prevalence of hydronephrosis in rats is low and usually does not exceed 5%–6% (Seely et al., 2018; Van Winkle et al., 1988), although there are published results demonstrating that the prevalence of hydronephrosis in a colony of healthy rats was nearly 70% (Arnold et al., 2011). Our results demonstrate that the percentage of hydronephrotic animals that were provided as healthy animals can reach up to 30%. Equivalent prevalence of hydronephrosis in rats has been documented after selective inbreeding of hydronephrotic animals in two generations (Van Winkle et al., 1988). Thus, these results indicate that a high portion of animals may be affected, which could affect the results or even lead to false results. Interestingly, two studies have shown the seasonality of hydronephrosis in rats and concluded that the incidence of hydronephrotic rats was decreased in animals born in autumn and winter (O'donoghue & Wilson, 1977; Van Winkle et al., 1988). Our results contradict these previously mentioned studies because animals in both groups were born in winter or early spring.

To characterize the kidney structure, we used ultrasonography, which is a safe and reliable method to diagnose hydronephrosis and can even be used in awake animals (Wang et al., 2007). Ultrasonographic analysis of the renal pelvis allows the detection of even the smallest dilatation (<10% of the area of kidney transverse plain), which can be unnoticed in postmortem examination, as the renal pelvis may collapse after the sectioning of the kidney. Interestingly, the size of the dilated renal pelvis did not change over the 8‐week observation period; thus, it can be concluded that the hydronephrosis‐inducing factor is present only in early development and disappears in later life, although the reason for the development of hydronephrosis is still unclear.

The renal I‐R model with different modifications is a frequently used experimental model to search for novel renoprotective therapies (Wei & Dong, 2012); however, to our knowledge, there is no published study in which the kidneys have been examined using ultrasonography or any other visual diagnostic method before inducing ischemia. Our results showed that rats with unilateral hydronephrosis after renal I‐R had higher concentration of kidney injury markers (KIM‐1 and creatinine) in urine than their healthy littermates. KIM‐1 is one of the most sensitive indicators of renal damage and it has been shown that urinary KIM‐1 concentration strongly correlates with the degree of renal injury after ischemia‐reperfusion (Vaidya et al., 2010). The analysis of traditional renal injury blood markers showed that there were no differences in BUN and creatinine concentrations in plasma. It can be explained that both blood markers are less sensitive and specific (Han et al., 2002; Vaidya et al., 2010) and if the differences in the degree of renal damage is not very prominent both markers are too insensitive to show the difference. In addition, it has been shown that there is no correlation between degree of renal damage and BUN or serum creatinine (Vaidya et al., 2010). Further experiments showed that I‐R induced significantly higher gene expression levels of kidney injury markers as well as more pronounced injury of renal tubules of the hydronephrotic kidneys than the healthy kidneys. Thus, it can be concluded that the elevated kidney injury markers in urine were due to the hydronephrotic kidneys. Therefore, animals with hydronephrosis should be excluded from studies because they would affect the results of the experiment. In addition, how treatment would interact with I‐R‐induced injury in the hydronephrotic kidneys is unpredictable. Excluding hydronephrotic animals would be very important if the unilateral renal I‐R model with the removal of contralateral kidney is exploited. Researchers have performed the above‐mentioned experimental model by removing the right (Zilberman‐Itskovich et al., 2019) or left (Ar'Rajab et al., 1994) kidney. As the right‐sided kidney is affected by hydronephrosis more often than the left‐sided kidney (Seely et al., 2018), groups that perform left nephrectomy with I‐R of the right kidney would obtain significantly higher levels of kidney injury and its markers in urine and plasma.

Microscopically, hydronephrosis is characterized by atrophy of the renal tubules, glomeruli, and pelvic urothelium (Zhou et al., 2003). Thus, the damage induced by hydronephrosis could alter renal function. Indeed, previously published results have shown that rats at the age of 10–18 weeks with bilateral hydronephrosis have increased serum creatinine levels, decreased creatinine clearance and increased 24‐hr urine volumes (Susic et al., 1975). Another study demonstrated significantly decreased inulin and para‐aminohippurate clearances in the hydronephrotic kidneys compared to the contralateral control kidneys. In addition, the same study showed significantly increased total urinary sodium and potassium excretion as well as increased fractional sodium and water excretion in the animals with congenital unilateral hydronephrosis compared to their littermates without hydronephrosis (Friedman et al., 1979). The results from our study revealed that rats with unilateral hydronephrosis at the age of 12–14 weeks and after an 8‐week observation period, which corresponds to an average experiment time, had similar BUN, creatinine clearance, creatinine concentration in the plasma and 24‐hr urine output to healthy animals. Thus, BUN and creatinine clearance cannot be used to distinguish animals with healthy kidneys from animals with unilateral hydronephrosis. In addition, we tested the effects of hydronephrosis on the pharmacokinetic profile of substances that are actively transported via the nephron tubular membrane. L‐carnitine is an endogenously synthesized molecule that is actively reabsorbed in the renal tubules via carnitine/organic cation transporter 2 (Stieger et al., 1995; Tamai et al., 1998), and furosemide is a synthetic diuretic that is actively secreted in the renal tubules via multidrug resistance‐associated protein 4 (Hasegawa et al., 2007). In the case of both studied substances, there were no significant differences in the clearance and AUC values between the groups. These results could probably be explained by the significant capacity reserve of the left healthy kidney (Barai et al., 2010), although we administered high doses of both substances. Moreover another explanation could be that there should be very pronounced degradation of renal parenchymal tissues to observe a decrease in renal function.

Our results demonstrate that hydronephrotic kidneys are more sensitive to I‐R‐induced damage than healthy kidneys. Interestingly, previous studies have shown that hydronephrosis can also affect the function of other organs and promote the development of different diseases. It has been claimed that due to urine stasis, hydronephrotic kidneys are more susceptible to infection than healthy kidneys, and pyelonephritis is a common sequela in hydronephrotic animals (Seely et al., 2018). Marsh et al. demonstrated that the presence of hydronephrosis was associated with left ventricular dysfunction in Zucker diabetic fatty rats (Marsh et al., 2007). Moreover a more recent study showed that congenital bilateral hydronephrosis in rats altered autonomic heart control (Arnold et al., 2011). In addition, it has been demonstrated that hydronephrotic rats are more sensitive to a high salt diet and develop hypertension faster than their healthy counterparts (Carlström et al., 2006). More importantly, experimental evidence has shown that the presence of hydronephrosis can affect the way blood vessels respond to treatment (Stieger et al., 1995). Thus, the status of the kidneys should be examined not only before studying kidney pathologies but also in experimental models of cardiovascular pathologies.

In conclusion, our results demo

nstrate that I‐R induces more damage to hydronephrotic kidneys than to healthy kidneys, and kidneys must be examined by ultrasonography or other visual diagnostic methods before experiments to exclude hydronephrotic animals to avoid obtaining imprecise results and making improper conclusions. Unilateral hydronephrosis does not affect the pharmacokinetics of substances that are transported via the nephron tubular membrane.

## CONFLICTS OF INTEREST

The authors declare no conflicts of interest. In addition, our aim was to draw the attention of the scientific community to the finding that even healthy animals provided by certified animal breeders should be examined before the experiments.

## AUTHOR CONTRIBUTION

R.V. and E.L. participated in research design. R.V., K.V., M.V., H.C., and O.Z.‐M. conducted the experiments. E.S. performed UPLC‐MS‐MS analysis. R.V., K.V., and M.V. performed data analysis. R.V., E.L. K.V., M.V., and M.D. wrote or contributed to the writing of the manuscript.

## ETHICAL STATEMENT

The experimental procedures were performed in accordance with the guidelines of the European Community as well as local laws and policies, and the procedures were approved by the Latvian Animal Protection Ethical Committee of the Food and Veterinary Service, Riga, Latvia.

## Supporting information



Table S1
