# Supplementary material for: Rats with congenital hydronephrosis show increased susceptibility to renal ischemia‐reperfusion injury
Source: Physiol Rep. 2020 Nov 18;8(22):e14638. doi: 10.14814/phy2.14638 (PMC7673629; doi:10.14814/phy2.14638)
Supplement: Supplementary file 1 — Table S1 [file PHY2-8-e14638-s001.docx]

## Supplementary data

Table 1 Gene accession number and primer sequences of qPCR primers

| **Gene symbol** | **Full name** | | **NCBI Accession number** | | **Forward primer sequence** (5'->3') | | | **Reverse primer sequence (5'->3')** | **Amplicon length, b** | |  |
| --- | --- | --- | --- | --- | --- | --- | --- | --- | --- | --- | --- |
| NGAL | Neutrophil gelatinase-associated lipocalin | | [NM_130741.1](https://www.ncbi.nlm.nih.gov/entrez/viewer.fcgi?db=nucleotide&id=18543344) | | GCGATTCGTCAGCTTTGCC | | | AATGCATTGGTCGGTGGGAA | 79 | |  |
| KIM1 | Kidney Injury Molecule 1 | | [NM_173149.2](https://www.ncbi.nlm.nih.gov/entrez/viewer.fcgi?db=nucleotide&id=402692443) | | CTCCATCATATACTCCTGCAGAC | | | GAAGCCCTTAGTCGGGTTTCTC | 119 | |  |
| S100a9 | Calprotectin (Calgranulin-B) | | [NM_053587.1](https://www.ncbi.nlm.nih.gov/entrez/viewer.fcgi?db=nucleotide&id=16758363) | | GGCACGAGCTCCTTAGCTTT | | | TCAGGGTGTCAGGATGTCCATA | 130 | |  |
| VCP | | Valosin-containing protein | | [NM_053864.2](https://www.ncbi.nlm.nih.gov/entrez/viewer.fcgi?db=nucleotide&id=40254739) | | AATATTTGACAAGGCACGACAAG | CCGGTTGGTAGCTCCAATGAT | | | 193 | |
